# Bispectral Index™ variation during living donor liver transplantation in a child with hepatic encephalopathy: a case report

**DOI:** 10.1186/s40981-021-00469-1

**Published:** 2021-08-28

**Authors:** Nozomu Yamada, Juntaro Shiba, Hiroaki Kikuchi, Takashi Yamada, Naoyuki Taga, Mamoru Takeuchi

**Affiliations:** Department of Anesthesiology and Critical Care Medicine, Jichi Medical University, 3311-1 Yakushiji, Shimotsuke-City, Tochigi, 329-0498 Japan

**Keywords:** Bispectral Index™, Pediatric acute liver failure, Hepatic encephalopathy, Liver transplantation

## Abstract

**Background:**

It is unclear whether perioperative Bispectral Index™ (BIS) monitoring in pediatric cases with acute liver failure (ALF) is effective for evaluation of neurological function. We describe a pediatric patient with hepatic encephalopathy (HE) in whom the BIS value increased from low levels to the normal range during liver transplantation (LT).

**Case presentation:**

Electroencephalography in a 6-year-old comatose girl diagnosed with ALF and HE who was unresponsive to pain and auditory stimuli revealed continuous slow waves, and hence, emergency LT was performed. Intraoperatively, BIS values remained low until reperfusion. However, BIS value variability increased after reperfusion. She was subsequently discharged without any neurological sequelae.

**Conclusions:**

Low BIS values were considered to reflect the severity of HE. It is possible that improvement of the BIS value and waveform was a reflection of graft function. BIS monitoring might be a good indicator of neurological recovery after LT.

## Background

Pediatric acute liver failure (ALF) is a life-threatening disease that requires rapid and appropriate intensive care [1]. In pediatric patients with ALF, it is difficult to assess the severity of disturbance of consciousness, which might delay the start of intensive care, including liver transplantation (LT) [2]. Furthermore, some cases might be contraindicated for LT due to irreversible central nervous system damage. Delayed LT might also lead to persistent neurological sequelae in some cases.

Several monitoring devices have been used to monitor consciousness. The Bispectral Index™ (BIS) monitor, a simple noninvasive device based on electroencephalographic parameters, is frequently used to evaluate the level of consciousness during general anesthesia. However, there is no report describing changes in BIS levels during emergency LT in pediatric patients with hepatic encephalopathy (HE). This case report suggests that BIS variations during LT for severe ALF with HE might be useful for predicting post-transplant neurological outcomes.

## Case presentation

A 6-year-old girl (height 110 cm, weight 20.3 kg) with no medical history had a fever 1 month before surgery. She visited a clinic and general hospital due to jaundice and malaise. At admission to the general hospital 2 weeks before surgery, she was diagnosed with acute hepatitis and was given liver protection drugs (Stronger Neo-Minophagen C, Lactulose, Vitamin K) and fresh frozen plasma, although her liver and coagulation function deteriorated during the course of treatment, along with decrease in platelet count. She was transferred to the pediatric intensive care unit (PICU) of our children's medical center for blood purification therapy 4 days before surgery.

On examination, she looked ill. Physical examination revealed the following: blood pressure 95/56 mm Hg, pulse rate 72 beats/min, respiratory rate 20 breaths/min with SpO_2_ of 99% on room air, body temperature 37.3 °C, and Glasgow Coma Scale (GCS) score of E3V4M6. Abdominal examination was unremarkable, with no hepatosplenomegaly. Examination of the eyes and skin revealed icterus.

**Fig. 1:**
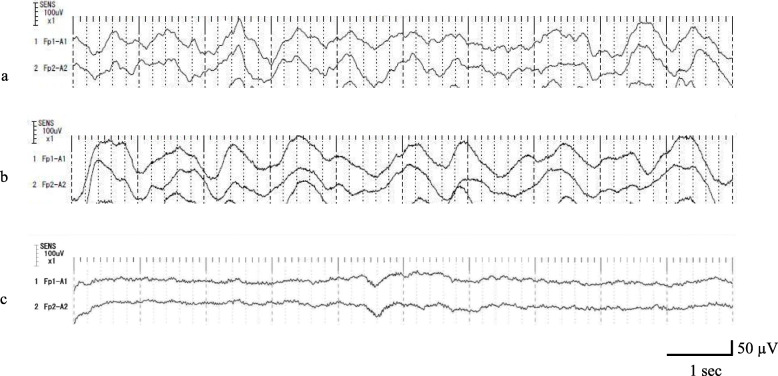
**a**–**c** Patient’s electroencephalograms during the course of treatment. **a** On admission to the PICU, showing slow spindle waves (1–2 Hz) without laterality and triphasic waves. **b** One day before surgery, showing high voltage continuous slow waves (0.5–1 Hz) without laterality. **c** On postoperative day 5, at rest with the eyes closed, showing low amplitude and fast waves (9–10 Hz) without laterality or paroxysmal waves. Fp1-A1 and Fp2-A2: left and right front polar-auricular. Only representative EEG waves are presented, because there were no remarkable changes in specific leads

Initial laboratory investigations revealed thrombocytopenia (hemoglobin 13.0 g/dL, white cell count 3.4 × 10^3^/μL, platelet count 9.1 × 10^4^/μL). Electrolytes were normal (Na 139 mmol/L, K 4.2 mmol/L, Cl 103 mmol/L, Ca 9.5 mg/dL, Mg 2.3 mg/dL, P 3.9 mg/dL). Her coagulation profile was prolonged (prothrombin time 23%, prothrombin time-international normalized ratio 2.36, activated partial thromboplastin time 63.7 s) and liver function tests were abnormal (albumin 4.0 g/dL, total bilirubin 18.76 mg/dL, direct bilirubin 13.17 mg/dL, venous ammonia 70 μg/dL, lactate 2.2 mmol/L). Infectious disease screening tests were negative. Computed tomography (CT) scan of her abdomen showed lower absorption in the liver compared to the spleen, suggesting ALF. Cranial CT scan and fundus examination were normal. Electroencephalogram (EEG) showed slow spindle waves with a frequency of 1–2 Hz, without laterality or triphasic waves (Fig. 1a). Daily plasma exchange and continuous hemodiafiltration were started for ALF with HE. Mannitol was administered to prevent cerebral edema. A day before surgery, GCS scores worsened to E1VTM1. Clinically, she was unresponsive to pain and auditory stimuli, and her EEG showed high voltage continuous slow waves with a frequency of 0.5–1 Hz, without lateralization (Fig. 1b). Assuming deterioration of HE, emergency LT was considered vital. After obtaining informed consent from her family, urgent living donor LT with an extended left liver graft from her mother was planned on the 5th ICU day.

**Fig. 2:**
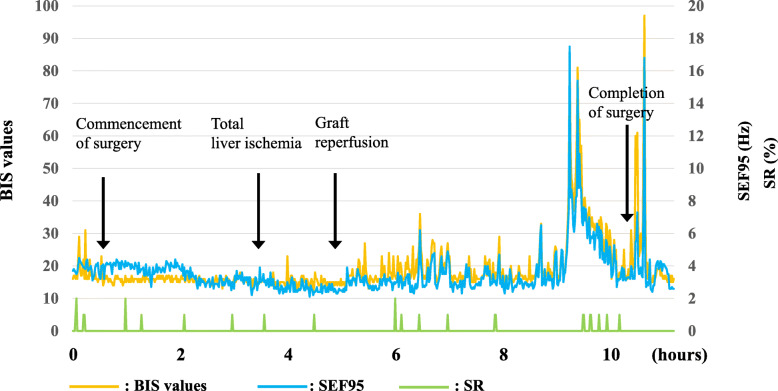
Trends in BIS values, SEF95, and SR during liver transplantation. Time 0 indicates anesthesia induction. The orange, blue, and green lines represent BIS values, SEF95, and SR, respectively. The graph of BIS values and SEF95 showed almost the same changes. BIS values ranged from 10 to 20 and 10 to 80 before and after reperfusion, respectively. SEF95 ranged from 2 to 4 Hz and 3 to 17 Hz before and after reperfusion, respectively. SR was 0–2% before and after reperfusion

**Fig. 3:**
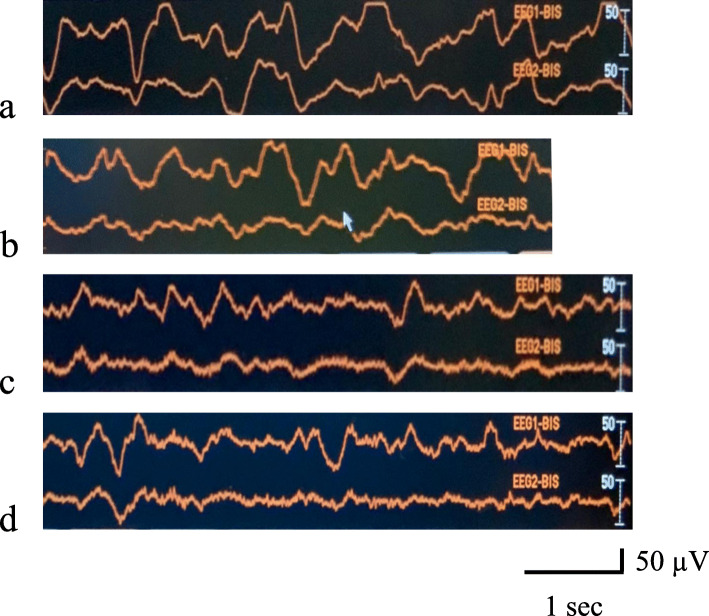
**a**–**d** Electroencephalogram recorded by the BIS monitor at each phase of liver transplantation. EEG1-BIS and EEG2-BIS were obtained from between the middle of the forehead and above the left eyebrow, and between the middle of the forehead and the left pre-auricular area, respectively. **a** The BIS value was 15 with high voltage and slow waves (0.5–1 Hz) in the dissection-anhepatic phase. **b** The BIS value was 24 with high voltage and slow waves (0.5–1 Hz) in the reperfusion phase. **c** The BIS value was 38 with moderate amplitude and slow waves (2–3 Hz) in the neo-hepatic phase. **d** The BIS value was 55 with moderate amplitude and slow waves (2–4 Hz) at the time of wound closure

General anesthesia was induced with fentanyl (50 mcg) and rocuronium (20 mg) and maintained with remifentanil and rocuronium infusions (0.1–0.4 mcg/kg/min and 7 mcg/kg/min, respectively), along with sevoflurane inhalation at 0.5%. A BIS Quatro Brain Monitoring Sensor (Medtronic plc, Dublin, Ireland) was placed on the frontotemporal region, and her electroencephalogram, including BIS values, 95% spectral edge frequency (SEF95), and suppression ratio (SR), was continuously recorded at each phase of liver transplantation by the BIS monitor until the end of anesthesia (Fig. 2, Fig. 3a–d). The durations of anesthesia and surgery were 708 min and 637 min, respectively. The graft volume was 254 g, graft volume/recipient standard liver volume (GV/SLV) was 46.1%, and graft-to-recipient weight ratio (GRWR) was 1.27%. Warm and cold ischemia times were 40 min and 51 min, respectively. BIS values ranged from 10 to 20 from the start of the operation to reperfusion (Fig. 2). From reperfusion to the end of the operation, BIS values ranged from 10 to 80, and the variation in BIS values also increased. The frequency of EEG waveforms and BIS values seen with BIS monitoring gradually increased (Fig. 2). She was re-admitted to the PICU after surgery, extubated on postoperative day (POD) 2 and discharged from the PICU on POD 7. Follow-up EEG on POD 5 showed low amplitude and fast waves with a frequency of 9–10 Hz, without laterality or paroxysmal waves (Fig. 1c). However, she spoke only a few words until POD 7. Two weeks postoperatively, she was newly diagnosed with hepatitis-associated aplastic anemia. Her immunosuppressant regimen was changed and continued for post liver transplantation prophylaxis and aplastic anemia. She was discharged after 4 months of hospitalization. The cause of liver failure in the present case remains unknown.

## Discussion

The BIS monitor is a non-invasive and simple device that digitizes the level of consciousness, and that was originally used to monitor the depth of general anesthesia. Previous reports have described increased clinical indications of BIS monitoring, including for assessing the severity of encephalopathy in adults [3, 4]. In this case, the BIS monitor was employed as a neurological monitor rather than anesthesia monitor, because the patient’s preoperative EEG showed an abnormal pattern and her consciousness had already deteriorated due to ALF and might have worsened during surgery. This case is the first report of evaluation of the severity of hepatic encephalopathy in a child using variability in BIS values during LT.

BIS values in the range of 40–60 are recommended as indicators of adequate depth of general anesthesia. Furthermore, it was reported that the calculated minimum alveolar concentration of sevoflurane for maintaining BIS values below 50 in children is 2.83%, which is three times as high as that in adults [5]. BIS values in our patient were as low as 10–20 until graft reperfusion, despite administration of 0.5% sevoflurane (Fig. 2). Additionally, the BIS monitor detected continuous moderate to high amplitude slow waves (Fig. 3a). This was considered to reflect the severity of HE [4]. Since rocuronium was administered continuously as a bolus, the effect of electromyography on BIS monitoring was excluded. Previous reports have suggested a good correlation between BIS values and SEF95, a parameter derived from power spectral analysis [6, 7]. The trends in BIS values and SEF95 (Fig. 3) in our patient were similar to those in previous reports. SEF95 ranged from 2 to 4 Hz from the start of surgery to reperfusion, and from 3 to 17 Hz from reperfusion to the end of the operation. Moreover, it has been reported that 0.8–2.3% sevoflurane leads to a dose-dependent decrease in SEF95, but not BIS values [7]. Hence, the BIS value was considered to be as reliable as SEF95 because of administration of 0.5% sevoflurane in this case. A correlation between BIS values and SR has also been reported [8]. However, as shown in Fig. 3, SR did not change in our case despite the increasing variation in BIS values after reperfusion in our patient, suggesting that SR does not appear to correlate with hepatic metabolism.

A previous report suggested that BIS increase after reperfusion indicates the return of cerebral activity with the restoration of graft hepatic function [9]. The report also described that BIS values increased after reperfusion in both living-donor LT and orthotopic LT groups. We considered that BIS changes in our case also reflected graft function. Moreover, another report suggested that BIS could be used to assess recovery of consciousness in patients with HE during peritransplant intensive care [10]. On the other hand, another report declared no correlation between BIS and postoperative neurological outcomes [4]. One case report reported dramatic BIS changes along with recovery of consciousness perioperatively in an adult patient with ALF who had altered mental function caused by HE [11]. Our pediatric patient demonstrated large BIS variations and waveform improvement after reperfusion (Fig. 3c, d). Lack of postoperative neurological sequelae in our patient suggests that intraoperative BIS recovery might predict postoperative neurological function even in pediatric cases. Overall, the perioperative management of ALF and LT was successful in our patient.

Pediatric ALF is a complex, rapidly progressive clinical syndrome that leads to severe multisystem organ failure and unpredictable and irreversible complications. It requires urgent decision-making about LT, which is the only proven treatment for pediatric ALF [1]. There is no scoring system that is adequate to support decisions about LT in pediatric ALF patients [2]. Additionally, there are still no optimal prognostic indicators in pediatric ALF. In our case, LT decision-making was based on repeated EEG, and the BIS monitor was employed as a simple EEG monitor during surgery, which showed the trend of BISs values during LT. Our experience suggests that EEG variations might predict neurological outcomes after LT in pediatric patients with ALF and HE.

## Data Availability

Not applicable.

## Abbreviations

ALF: Acute liver failure
BIS: Bispectral index™
CT: Computed tomography
EEG: Electroencephalogram
GRWR: Graft-to-recipient weight ratio
GV/SLV: Graft volume/recipient standard liver volume
HE: Hepatic encephalopathy
LT: Liver transplantation
MRI: Magnetic resonance imaging
PICU: Pediatric intensive care unit
POD: Postoperative day
SEF95: 95% Spectral edge frequency
SR: Suppression ratio
